# Diagnostic and Prognostic Indications of Nasopharyngeal Carcinoma

**DOI:** 10.3390/diagnostics10090611

**Published:** 2020-08-19

**Authors:** Engku Nur Syafirah E. A. R., Ahmad Adebayo Irekeola, Chan Yean Yean

**Affiliations:** 1 Department of Medical Microbiology and Parasitology, School of Medical Sciences, Universiti Sains Malaysia, Health Campus, Kubang Kerian 16150, Kelantan, Malaysia; engkunursyafirah@gmail.com (E.N.S.E.A.R.); profahmad007@yahoo.com (A.A.I.); 2 Department of Biological Sciences, Microbiology Unit, College of Natural and Applied Sciences, Summit University Offa, Offa PMB 4412, Kwara State, Nigeria; 3 Hospital Universiti Sains Malaysia, Universiti Sains Malaysia, Health Campus, Kubang Kerian 16150, Kelantan, Malaysia

**Keywords:** tumor-suppressor gene, oncogene, miRNA, nasopharyngeal carcinoma, tumor biomarker

## Abstract

Nasopharyngeal carcinoma (NPC) is a disease that is highly associated with the latent infection of Epstein–Barr virus. The absence of obvious clinical signs at the early stage of the disease has made early diagnosis practically impossible, thereby promoting the establishment and progression of the disease. To enhance the stride for a reliable and less invasive tool for the diagnosis and prognosis of NPC, we synopsize biomarkers belonging to the two most implicated biological domains (oncogenes and tumor suppressors) in NPC disease. Since no single biomarker is sufficient for diagnosis and prognosis, coupled with the fact that the known established methods such as methylation-specific polymerase chain reaction (PCR), multiplex methylation-specific PCR, microarray assays, etc., can only accommodate a few biomarkers, we propose a 10-biomarker panel (KIT, LMP1, PIKC3A, miR-141, and miR-18a/b (oncogenic) and p16, RASSF1A, DAP-kinase, miR-9, and miR-26a (tumor suppressors)) based on their diagnostic and prognostic values. This marker set could be explored in a multilevel or single unified assay for the diagnosis and prognosis of NPC. If carefully harnessed and standardized, it is hoped that the proposed marker set would help transform the diagnostic and prognostic realm of NPC, and ultimately, help prevent the life-threatening late-stage NPC disease.

## 1. Introduction

Nasopharyngeal carcinoma (NPC) is a malignant, undifferentiated squamous cell carcinoma, which is highly associated with latent infection of Epstein–Barr virus (EBV) [1]. EBV infection is highly prevalent; ubiquitously infecting people around the globe and mostly go asymptomatic [2]. Although NPC constitutes a major health burden in Southern China, Southeast Asia, the Arctic, and the Middle East/North Africa regions [3], to date, the exact cause of the disease is still unclear. However, high-risk factors that may contribute to NPC include EBV infection, dietary habits and lifestyle, cigarette smoking, exposure to environmental and occupational hazards, genetic predisposition (particularly chromosomal regions and genes), family history of NPC, and ethnicity [4].

The clinical presentation of NPC varies, ranging from unspecific epistaxis, auditory complaints, and unilateral nasal obstruction to cranial nerve palsies and nodal metastasis in the neck region [5,6]. Most people diagnosed with NPC are often at mid or late stages of the disease when symptoms are much obvious [7]. This underscores the need for early diagnosis. Sadly, the diagnosis of NPC at an early stage is quite challenging due to its deep location and lack of obvious clinical signs at an early stage [8]. Furthermore, there is no single laboratory blood test that provides a highly sensitive and specific result for the screening and diagnosis of NPC [9]. The consequence of poor test results cannot be overemphasized. Tests with low sensitivity could give rise to false-negative results, leading to missed diagnosis, and thus, promotion of disease progression. On the other hand, false-positive results can ensue from tests with low specificity, amounting to unnecessary nasoendoscopies, biopsy collection, and follow-up visits [9]. Even though the assessment of clinical symptoms and family history provides a good cue, nasopharyngeal endoscopy accompanied by histopathological examination of suspected lesions remains the gold standard method for NPC diagnosis [10]. However, this method is mainly applicable for suspected NPC patients and unsuitable for early diagnosis, especially in asymptomatic patients.

**Figure 1:**
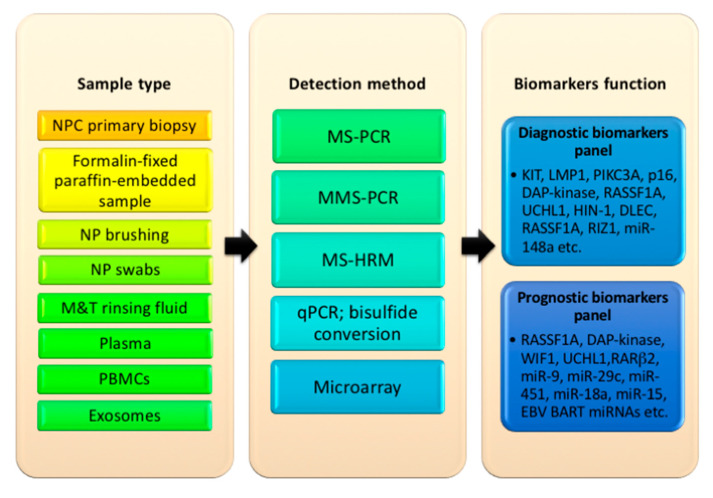
A summary of diagnostic and prognostic indications for the detection of nasopharyngeal carcinoma (NPC) using oncogenic and tumor suppressor biomarkers. NP: nasopharyngeal; M&T: mouth and throat; PBMCs: peripheral blood mononuclear cells; MS-PCR: methylation-specific polymerase chain reaction (PCR); MMS-PCR: multiplex methylation-specific PCR; MS-HRM: methylation-sensitive high-resolution melting; qPCR: real-time PCR; EBV: Epstein–Barr virus.

Given that tumorigenesis is a complex process stimulated by many factors, including environment (work hazard, physical exposure, microorganisms, etc.), genetics, and epigenetics [11], a multifaceted diagnostic approach may help address the challenges of early diagnosis. Notable mechanisms underlying tumorigenesis encompass epigenetic changes, genetic codes mutation, chromosome stability, DNA repair, and cell growth process (differentiation, apoptosis, movement, etc.) [12]. Deregulation of oncogenes and tumor suppressor genes (TSGs) stimulated by genetics and epigenetics is considered a driving force in the growth and progression of cancer [13]. For example, overexpression of c-Myc (an important oncogene) protein plays a role in the malfunctioning of several important cellular processes including cell growth control, proliferation, apoptosis, and cellular metabolism [14]. Similarly, the dysfunction of p53 (an important TSG) protein promotes cell propagation with serious DNA damage and implicated with interruption of cell cycle arrest and/or apoptosis (the p53-dependent apoptosis) [15]. However, in NPC, the oncogene–TSG regulatory relationship as it impacts tumorigenesis is not well understood. This is further compounded by the fact that EBV also expresses viral oncogenic genes (EBNA1 and LMP1) and miRNAs (BARTs and BHRF1) which can induce genetic mutations and epigenetic changes in host cells, consequently leading to tumorigenesis and progression of cancer [16]. Here, we summarize cellular and viral (EBV) protein and nonprotein coding oncogenic and tumor suppressor candidates, highlighting their potentials as diagnostic and prognostic tumor biomarkers in NPC (Figure 1).

## 2. Cellular Oncogenes Involved in NPC

Oncogene mutations, which contribute to tumorigenesis, may serve as diagnostic and prognostic targets for NPC. However, oncogenic mutation patterns in NPC are not fully elucidated compared with other cancer types [17]. A study by Jiang et al. identified 24 hotspot mutations across 11 oncogenes (EGFR, CDK4, KIT, PDGFRA, KRAS, BRAF, MET, FGFR3, AKT1, PIK3CA, and NRAS) in NPC patients and found that KIT mutation was associated with poorer overall and relapse-free survival [17]. Similarly, Zhang et al. found positive mutations in eight oncogenes (PIK3CA, NRAS, KIT, PDGFRA, ABL, HRAS, EGFR, and BRAF) in NPC tumors. Interestingly, patients with these mutations tend to experience relapse or metastasis [18]. A study was conducted to screen targeted therapy-related oncogenic mutations in NPC using SNaPshot assay. Among 70 patients, 12 harbored mutations in five oncogenes (KIT, EGFR, PIK3CA, KRAS, EGFR/BRAF) with KIT mutation being the most prevalent. In addition, a patient was found to have dual oncogenic mutations (EGFR and BRAF). However, there was no association between the observed oncogenic mutations and tumor recurrence and metastasis [19]. Meanwhile, Chai et al. reported a significantly high level of FJX1 expression in primary NPC tissues, and the overexpression of FJX1 was associated with the promotion of cell proliferation, anchorage-dependent growth, and cellular invasion in vitro [20].

### EBV-Associated Oncogenes in NPC

Apart from oncogenic mutations that occur in the human genome, the oncogenic human herpesvirus, EBV, is also closely associated with NPC development [1]. In the EBV genome, there are three potential NPC oncogenes—LMP1, LMP2, and BARF1 [21]. LMP1 is known as classical oncogenic protein and it is the main transforming protein of EBV [1]. LMP2 on the other hand can transform epithelial cells in vitro, while BARF1 is known for its oncogenic properties in NPC [22,23]. Hu et al. reported the expression of these putative oncogenes (LMP1, LMP2, and BARF1) in the majority of NPC samples compared with normal samples [21]. Wang et al. also reported the expression of LMP1 and BARF1 in tissue specimens of NPC patients and revealed an association between the expression of LMP1 and tumor-node-metastasis stage as well as lymph node metastasis [24]. However, in another study, the abundance of LMP1 protein is not correlated to the presence of lymph node or visceral metastasis or recurrence in north African NPC patients [25].

## 3. Hypermethylation of TSGs Promoter in NPC

Several studies have proposed that loss of heterozygosity (LOH) on chromosome 3p, 9p, 11q, and 13q regions are an early event for tumorigenesis in NPC [26,27,28,29]. LOH of the 3p chromosome region has been shown in about 95–100% of NPC cases and 75% of premalignant lesions [28]. These studies have suggested that aberrant hypermethylation at the promoter region of CpG islands and genetic alterations in chromosome underlie the development and progression of NPC. Furthermore, rising evidence reveals that multiple hypermethylated promoter regions are present in NPC epithelial cells (Table 1).

An attempt was made in the past to identify hypermethylation of p16 promoter in NPC using xenograft, cell line, and primary tumor [30]. Subsequently, few other studies were performed using methylation-specific polymerase chain reaction (MS-PCR) targeting p16. Approximately 23–66% of p16 methylation frequencies were recorded in primary undifferentiated NPC, followed by 46.4% in nasopharyngeal brushings and 42% in plasma [31,32,33,34,35]. A recent meta-analysis study has also suggested that p16 promoter hypermethylation significantly increases the risk of getting NPC in the future [30,36]. The results suggest that p16 promoter hypermethylation could be used as a potential diagnostic target for differentiating nonmalignant from malignant nasopharyngeal tumors.

Hypermethylation of Ras Association Domain Family 1A (RASSF1A) promoter is another potential biomarker for early detection of NPC. Several reports showed that the methylation frequency of RASSF1A promoters can be as high as 46–91% in primary tumors, 39.3% in nasopharyngeal brushings, 33% in nasopharyngeal swabs, and 37% in mouth and throat-rinsing fluid [38,39,40,41]. Similar cases have been observed in Death-associated Protein Kinase (DAP-kinase) promoter hypermethylation where the methylation frequency of DAP-kinase promoters was as high as 75–77% in NPC primary tumors, 63% in nasopharyngeal swabs, 50% in both nasopharyngeal brushing and mouth and throat-rinsing fluid and 20% in plasma sample [35,38,43].

Other known TSGs promoter hypermethylation in NPC include DAPK1, DLEC1, CDKN2A, E-cadherin, p15, WIF1, UCHL1, PTEN, etc., which can be used as alternative surrogate markers for early NPC diagnosis. Nevertheless, a single epigenetic change is not sufficiently sensitive and accurate for early detection of NPC especially in tissue biopsies or body fluids. This is because patients are less likely to have the same TSG hypermethylation profile. Therefore, focusing on single epigenetic changes can lead to false-negative results in diagnosis. Thus, evaluating multiple specific gene methylation profiles have been suggested to improve the sensitivity of NPC diagnosis [44]. Nawaz et al. utilized multiplex methylation-specific PCR (MMSP) to screen 10 potential TSG markers for NPC diagnosis, including two markers from EBV. The study found that combined analyses of 10 methylation markers (RASSF1A, DAPK, ITGA9, p16, WNT7A, CHFR, CYB5R2, WIF1, RIZ1, FSTL1) and two EBV markers (EBNA1 and LMP1) provided good discrimination between NPC and NPC control tissues with a detection rate of 91% in biopsies with 90% specificity [44]. Another study that targeted the methylation of four TSGs (RASSF1A, WIF1, DAPK1, and RARβ2) alongside EBV DNA markers showed significant detection of NPC at all stages as well as local recurrence [45].

It has been demonstrated that the combination of four methylation gene markers (CDKN2A, DLEC1, DAPK1, and UCHL1) can be employed for the prediction of early NPC [32]. Furthermore, the combination of RASSF1A and p16 methylation markers give good discrimination between NPC and non-NPC samples, although better results could be obtained by combining five methylation markers (RASSF1A, p16, WIF1, CHFR, and RIZ1) [49]. The identification of multiple methylated TSGs has also been utilized as a prognostic cue in NPC. A study that investigated a panel of six hypermethylated genes (WIF1, UCHL1, RASSF1A, CCNA1, TP73, and SFRP1) in NPC revealed that high methylation level is associated with poor disease-free survival [42]. Overall, these data support the utilization of some specific hypermethylated TSGs as key indicators of NPC and could be targeted in the prediction, diagnosis, and prognosis of the disease.

## 4. Noncoding RNAs in NPC

The oncogenic and tumor-suppressive roles of noncoding RNA (ncRNA), especially the regulatory ncRNAs, have been explored in a vast majority of cancers. Many are now considered useful diagnostic, prognostic, and therapeutic biomarkers of cancers. NcRNAs (including short and long ncRNAs) have also been implicated in NPC. Widely studied and noteworthy ncRNA biomarkers mostly implicated in NPC include miRNA and long noncoding RNAs (lncRNAs). Others like small nucleolar RNA (SnoRNA), small interfering RNA (siRNA), Y RNA, etc., are rarely implicated in NPC. For instance, dysregulation in several SnoRNAs (e.g., snoRA21, snoRA23, snRD112-114, snoRD33, snoRD5, etc.) have been associated with many types of cancers including breast cancer, hepatocellular carcinoma, colorectal cancer, prostate cancer, acute promyelocytic leukemia, among others (reviewed in [52] and [53]), however, there is much quiescence regarding their role in NPC. Furthermore, only a few recent studies have demonstrated the upregulation of lncRNA small nucleolar RNA host genes (SNHGs) such as SNHG5 [54], SNHG7 [55,56], SNHG12 [57] and SNHG20 [58] in the blood and tissue samples of NPC patients. Thus, we focused on miRNA and long noncoding RNA for which there is more evidence to support the prognostic and diagnostic potentials in NPC, particularly using human subjects.

### 4.1. MicroRNA (miRNA or miR) in NPC

Although microRNAs (miRNAs) are known to be involved in the regulation of the cellular process, studies suggest that they may be associated with the manifestation of cancer [59]. MiRNAs also consist of tumor suppressor miRNAs and oncogenic miRNAs (onco-miRNAs). The loss-of-function of tumor suppressor miRNAs and gain-of-function of onco-miRNAs appears to enhance the formation of cancer [59]. The most common tumor suppressor miRNAs involved in NPC are miR-9, miR-26a, miR-29c, miR-200 family, and Let-7 family. The overexpression of onco-miRNAs miR-141, miR-214, miR-18a/b, miR-155, and miR-21 have also been identified in NPC [60,61,62] (Table 2).

Tumor suppressor miR-9 is one of the most implicated miRNAs in NPC [62]. The miR-9 functions to regulate essential cellular processes (proliferation, migration, apoptosis, etc.), metastasis, angiogenesis, and it is involved in epithelial–mesenchymal transition (EMT) through binding to the 3′-UTR of CXCR4 receptor to downregulate its expression [63]. However, the miR-9 expression in NPC is commonly downregulated, and low-level expressions of miR-9 in plasma are significantly associated with increased lymphatic invasion, aggressive phenotypes, advanced NPC stage, and poor survival rates [86]. Most importantly, miR-9 could be an independent prognostic biomarker for NPC metastasis stage as its high expression is correlated with decreased proliferation, migration and invasion in NPC cells, and low expression correlated with advanced NPC stage [87]. Onco-miRNA miR-18a/b on the other hand is a member of the oncogenic miR-17-92 cluster which plays a role in NPC development. MiR-18a is indeed upregulated in NPC samples and correlates with advanced NPC stages, metastasis in the lymph node, EBV infection, and higher mortality rates [61]. Meanwhile, miR-18b directly suppresses connective tissue growth factor (CTGF) expression in NPC which leads to NPC progression and poor prognosis due to the downregulation of CTGF [78].

Again, a single miRNA marker is not sufficiently sensitive and precise for prognosis or early detection of NPC due to the possibility of different miRNA profiles among patients. Thus, investigating multiple miRNA biomarker profiles would be beneficial to improve the sensitivity of NPC diagnosis and prognosis. It is important to note that circulating miRNAs have been identified as potential noninvasive biomarkers for diagnosis and prognosis in multiple cancers due to their remarkable stability in the blood [88]. A recent study that analyzed the changes in plasma miRNAs before and after treatment of NPC showed that miR-9-3p, miR-124-3p, miR-892b, and miR-3676-3p were expressively upregulated after treatment compared with pretreatment, and downregulated at recurrence phase or metastasis [86], highlighting the potential of miRNAs as biomarkers for monitoring recurrence and metastasis in NPC patients. In another study, it was found that four miRNAs (miR-17, miR-20a, miR-29c, and miR-223) were expressed differentially in the serum of NPC patients compared with noncancerous control [89]. Furthermore, a study advanced the combination of four serum miRNAs (miR-22, miR-572, miR-638, and miR-1234) as a potent miRNA signature to categorize high- and low-risk groups of NPC patients in terms of overall survival and distant metastasis-free survival [90].

After its discovery in 2001, miR-21 has been reported as associated with the development of cancer. It is, in addition, one of the most studied miRNAs in human cancers [76]. MiRNA-21 expression is also substantially increased in many cancers and may play a critical role in cancer cell survival, invasion, and apoptosis [76,91]. Recent findings suggest that miR-21 induced the expression of a significant number of cancer-related genes such as PDCD4, PTEN, SPRY, ERCK, TPM1, and Bcl-2, and responsible for regulating lymphocyte function [76,92]. MiR-21 inhibitors have shown suppression of NPC cell proliferation and migration by Bcl-2 downregulation [60]. The upregulation of circulating miR-21 may increase the chemoresistance of NPC cells to cisplatin and thus be associated with poor NPC prognosis [91]. The above studies bolster the assertion that loss-of-function of tumor suppressor miRNAs and gain-of-function of onco-miRNAs can be ascribed to NPC progression, advanced NPC stage, and poor survival rates.

### 4.2. EBV-Encoded BART miRNAs in NPC

Like cellular miRNA, studies have shown that EBV miRNAs may also be connected with the development of NPC [93]. Ye et al. demonstrated that EBV-miR-BARTs play a role in modulating metabolism-associated gene expression in NPC cells [94]. Expression of EBV-BART miRNAs in NPC contributes to numerous effects, including virus latency (BART2, BART6-5p), cell growth and proliferation (BART3, BART9, BART10, BART7, BART8), cell apoptosis (BART 1-5p, 16, 17-5p, BART cluster 1, BART5, BART20), as well as tumor metastasis and recurrence (BART1, BART7, BART9) [85].

The characterization of EBV miRNA transcriptome of clinical NPC tissues showed that most of the high copies of EBV miRNAs share seed sequences (2–7 nucleotides) with human miRNAs, suggesting that the content of seed sequence may be significant reason underlying the accumulation of EBV-encoded proteins, BART miRNAs [95]. Gourzones et al. found that miR-BART17 is significantly more abundant in plasma samples of NPC patients with a sensitivity of 77% and up to 90% specificity. Interestingly, they found a marked increase of miR-BART17 accompanied by an increase in tumor mass in one patient, suggesting that the concentration of miR-BART17 in plasma may be associated with NPC progression [96]. In addition, another study found that miR-BART7 is significantly higher in NPC patients and also enhanced proliferation, migration, and invasion of NPC cells in vitro [97]. Thus, miR-BART7 may be useful as a novel prognostic serological biomarker for predicting NPC treatment efficacy [98].

### 4.3. Long Noncoding RNAs (lncRNAs) in NPC

LncRNAs display oncogenic and tumor suppressor characteristics with varying tissue- or cell-specific gene expression profiles. LINC00312-NAG7 is one of the first lncRNAs studied and functions as a tumor suppressor gene in NPC [99]. LINC00312-NAG7 acts to reduce the spread of NPC cells and prevent the progression from G1 to S phase in the cell cycle, thus aggravating cell apoptosis. The LINC00312-NAG7 expression is negatively correlated with tumor size but positively correlated with lymph node metastasis [100]. LINC00312-NAG7 is also highly negatively associated with EBV-encoded noncoding RNA and EBER1 in NPC. However, LINC00312-NAG7 expression could be used to distinguish NPC and noncancerous cells [100].

LncRNA LINC01133 in chromosome 1q23.2 has been shown to be downregulated in colorectal cancer, gastric cancer, oral squamous cell carcinoma, and NPC [101,102,103,104]. In NPC, LINC01133 has been shown to inhibit cell proliferation, invasion, and migration, both in vitro and in vivo. LINC01133 was indicated to have tumor suppressor function in NPC as its expression was lower in NPC samples (*n* = 31) compared to normal samples (*n* = 10) [101]. On the other hand, LncRNA Plasmacytoma variant translocation 1 (PVT1) is an oncogene which shows higher expression in NPC cells than normal nasopharyngeal epithelial tissue (64%, 60/94 vs. 18%, 6/33; *p* < 0.001), and higher expression of PVT1 resulted in worse progression-free survival (*p* = 0.0028) and overall survival (*p* = 0.0006) [105]. Thus, these biomarkers could be useful diagnostic and prognostic indicators of NPC.

Other known oncogenic lncRNAs involved in NPC include HOTTIP, HOTAIR, ROR, XIST, HNF1A-AS, DRAIC, NPCCAT1, ANRIL, H19, LINC01385, LINC01503, CASC15, PXN-AS1-L, LINC00460, and UCA1, which involve in increasing cell proliferation, migration, invasion, and metastasis [106,107,108,109,110,111,112,113,114,115,116,117,118,119,120]. High expressions of LINC00319, LINC01503, HOTTIP, PXN-AS1-L, ANRIL, HOTAIR, LINC01385, and XIST are associated with poor prognosis and short overall survival in NPC [106,107,109,113,115,117,120,121]. LncRNAs, LINC00346, and ROR play a critical functional role in chemoresistance, particularly cisplastin therapy [108,122], while MALAT1, PVT1, and NEAT1 play a major role in radioresistance [105,123,124] (Table 3).

### 4.4. EBV-Encoded BART lncRNAs in NPC

Like cellular lncRNAs, studies have shown that EBV lncRNAs may also be associated with NPC development owing to their varied expressions in EBV-infected cells, exosomes, and EBV-associated cancers. A study showed that EBV-miR-BART6-3p can directly target and downregulate lncRNA LOC553103 [132]. Furthermore, overexpression of lncRNA LOC553103 can promote tumor spread, invasion, mesenchymal transition, and metastasis in vitro and in vivo [132]. BC200, LINC00672, and LINC00982 expression profiles have been shown to be linked to EBV-associated epithelial cancer cells, like NPC [133]. The findings showed substantial upregulation of BC200 and LINC00672 in EBV-infected 293 cells while LINC00982 was downregulated [133].

## 5. Types of Sample Used to Detect Oncogenes, Promoter Hypermethylation, and miRNAs in NPC

Currently, NPC diagnosis relies largely on the tissue-sampling method via endoscopic biopsy of suspected tumor sites. This rather invasive method is not suitable for the early detection of NPC. The need to explore liquid biopsies from peripheral blood (plasma/serum/peripheral blood mononuclear cells (PBMCs)/exosomes) is paramount since they also carry certain tumor biomarkers. Furthermore, liquid biopsy possesses remarkable benefits over the traditional solid biological tissue sampling; it has low risk, it is noninvasive and nearly painless, no surgery is required, and it reduces the cost and time of diagnosis [134]. In addition, nasopharyngeal brushing, nasopharyngeal swabs, and throat-rinsing fluids can also be used in NPC diagnosis due to their noninvasiveness and convenient tissue-sampling method rather than conventional tissue-sampling method [135]. However, the frequency of NPC biomarkers may vary in different sample types. We provide a summary of the major types of samples used in detecting oncogenes and tumor suppressors in NPC (Table 4) to ease and enhance decision on sample selection for future NPC studies.

## 6. Proposed Selection of Oncogene-Tumor Suppressor Biomarkers for Diagnosis and Prognosis in NPC

As delineated above, there are several potential oncogenes and tumor suppressors that could be further explored to develop a less invasive and dependable diagnostic assay for the early detection of NPC. Because the use of a single biomarker has been shown to be unreliable for the detection and prognosis of NPC, we recommend a multilevel screening or the development of an assay that would encompass these main identified biomarker domains (oncogene and tumor suppressors) to enhance better specificity in the detection of NPC. Based on the frequencies and diagnostic values demonstrated, we propose here a 10-biomarker panel for the duo: KIT, LMP1, PIKC3A, miR-141, and miR-18a/b (oncogenic biomarkers) and p16, RASSF1A, DAP-kinase, miR-9 and miR-26a (tumor suppressors). It is hoped that these biomarkers, if properly harnessed and standardized, would enable a more conclusive and dependable NPC diagnosis. If carefully exploited, we strongly believe they can as well serve as potent indicators in the prognosis and determination of the metastatic stage of NPC.

Methods that could be used for the detection of tumor biomarkers include methylation-specific PCR, multiplex methylation-specific PCR, methylation-sensitive high-resolution melting, real-time quantitative PCR after bisulfide conversion, and microarray assays [15,16,29,32,35,53]. However, these methods favor the selection of only a few markers. Thus, our proposed streamlined biomarker set can be better investigated. In any case, to assess the diagnostic strength of these select biomarkers, it is imperative to measure the result against the gold standard detection method to establish reliability. In addition to this, the biomarker profile must be assessed before and after treatment for the evaluation of prognostic potential. Finally, large scale studies using different sample types would be required for the validation of these NPC biomarker sets.

## 7. Conclusions

NPC is a disease whose exact cause remains largely unknown. Its developmental process is complex and has been attributed to genetics and epigenetics, as well as biological and environmental factors (e.g., work hazard, physical exposure, microorganisms, etc.). In this review, oncogenes, tumor suppressor genes, and microRNA of diagnostic and prognostic potentials in NPC were discussed. Furthermore, we proposed a 10-biomarker set (KIT, LMP1, PIKC3A, miR-141, and miR-18a/b (oncogenic biomarkers) and p16, RASSF1A, DAP-kinase, miR-9, and miR-26a (tumor suppressors)) that could be focused upon and further explored in future studies. It is hoped that these select biomarkers would enhance the development of a reliable diagnostic and prognostic NPC tool.

## Figures and Tables

**Table 1:** Summary of hypermethylated tumor suppressor genes (TSGs) involved in NPC.

TSG Biomarkers	Main Biological Function(s)	Method of Detection	Function of Biomarkers	Reference
p16	Inhibitor of cyclin-dependent kinases (CDK) which slows down the cell cycle by hindering progression from G1 phase to S phase	MS-PCR	Diagnosis	[30,31,32,33,34,35,36,37]
p15	Inhibits the growth of some kinds of tumor cells and acts as a mediator of TGF-β induced cell arrest. p15 shares extensive homology with p16	MS-PCR	Diagnosis	[38,39]
RASSF1A	Regulates microtubule dynamics, cell cycle progression, and apoptosis	MS-PCR	Diagnosis, prognosis	[38,39,40,41,42]
DAP-kinase	Activates in various cellular activities including regulation of apoptosis, caspase-dependent death programs, cytoskeletal dynamics, and immune functions	MS-PCR	Diagnosis	[32,35,38,43,44,45]
CDH1	Involved in various mechanisms like regulating cell-to-cell adhesions, mobility and proliferation of epithelial cells especially E-cadherin protein	MS-PCR	Diagnosis	[31,39,46]
E-cadherin	Important mediators of cell-to-cell interactions in epithelial tissues and holds cells together	MS-PCR	Diagnosis	[38,47]
HIN-1	Inhibitor for cell growth, invasion, and AKT1 activation	MS-PCR	Diagnosis	[48]
MGMT	Has the ability to stoichiometrically repair DNA adducts and to self-inactivate	MS-PCR	Diagnosis	[39]
MLH1	Provides instructions for making a protein which plays an important role in DNA repairs by fixing errors during DNA replication in preparation for cell division	MS-PCR	Diagnosis	[39]
RIZ1	Induces G2-M cell cycle arrest and/or apoptosis	MS-PCR	Diagnosis	[44,49]
DLEC1	Suppresses tumor growth and reduces the invasiveness of cancer cells	MS-PCR	Diagnosis	[32]
UCHL1	Provides instructions for making ubiquitin carboxyl-terminal esterase L1 enzyme which is involved in cell machinery that degrades unwanted proteins	MS-PCR	Diagnosis	[32,42]
WIF1	A lipid-binding protein which binds to Wnt proteins and prevents them from triggering signaling pathways	MS-PCR	Diagnosis, prognosis	[42,44,49]
DLC1	Has the ability to enhance activated GTP-bound Rho-GTPases’ intrinsic ability to convert their GTP into GDP, thus rendering them inactive	MS-PCR	Diagnosis	[49]
SOX11	Regulates embryonic development and determines the cell’s fate	MS-PCR	Diagnosis, prognosis	[50]
14-3-3 sigma	Act as a negative regulator in the cell cycle and has been identified as p53-inducible gene product involved in cell cycle checkpoint control after DNA damage	MS-PCR	Prognosis	[51]

MS-PCR; methylation-specific polymerase chain reaction.

**Table 2:** Summary of tumor suppressor miRNAs and onco-miRNAs involved in NPC.

MiRNAS (Biomarkers)	Main Biological Function(s)	Validated Targets in NPC	Prognostic Association
**Tumor Suppressor MiRNAs**			
miR-9	Regulates cell proliferation, migration, invasion, epithelial–mesenchymal transition (EMT), metastasis, apoptosis, and tumor angiogenesis	CXCR4 [63]	Negative
miR-26a	Suppress cell proliferation and colony formation	EZH2 [64,65], c-Myc [61]	-
miR-29c	Inhibits cell migration and invasion, metastasis, associated with chemoresistance and radioresistance	TIAM1 [66], MCL-1 [67], BCL-2 [67]	Negative
miR-200 family	Regulates cell proliferation, migration, invasion, and EMT	ZEB2 [68], CTNNB1 [68]	-
Let-7 family	Inhibits cell proliferation and induces cell apoptosis	c-Myc [69], HMG2A [70]	-
miR-124	Inhibits cell growth, migration, and invasion	Foxq1 [71]	-
miR-451	Regulates cell proliferation, invasion and predicts outcome	MIF [72]	Negative
miR-216b	Suppress cell proliferation and invasion	PKCa [73], K-Ras [74]	-
miR-98	Suppress NPC relapse and predicts recurrence	EZH2 [65]	Negative
miR-375	Suppress NPC relapse and predicts recurrence	Metadherin [75]	Negative
**Onco-miRNAs**			
miR-21	Promotes cell proliferation and migration and high expression resulting in chemoresistance	PTEN/AKT [76], PDCD4 [76], TPM1, SPRY [76], ERCK [76], Bcl-2 [60]	Positive
miR-18a	Lymph node metastasis, overall downregulation of miRNA expression	Dicer1 [77], c-Jun and c-Myc [77]	Positive
miR-18b	Promotes cell proliferation	CTGF [78],	-
miR-141	Promotes cell proliferation, migration, invasion, and cell apoptosis	BRD3 [79], PTEN [79], SPLUNC1 [79], UBAP1 [79]	-
miR-214	Promotes cell proliferation, invasion, and metastasis	Lactotransferrin [80]	-
miR-30a	Increase cell capability of metastasis and invasion	E-cadherin [81]	-
miR-149	Promotes cell migration, EMT, and invasion	E-cadherin [82]	-
miR-155	Promotes cell proliferation, migration, invasion, colony formation, invasion, prognostic for tumor stage and predicts outcome	JMJD1A [83], BACH1 [83]	Positive
miR-504	Predicts radioresistance outcome	NRF1 [84]	Positive
EBV-encoded BART miRNAs such as BART 1-3p, 5p, BART5, BART 6-5p, BART 7, BART 3, -6, -8, -16, -22	Promotes tumor metastasis, cellular growth, and proliferation, inhibit cell apoptosis, and maintain virus latency	PTEN [85], PUMA [85], DICE1 [85], E-cadherin [85], Dicer [85], C-myc, and C-jun [85]	Positive

Prognostic association: positive; higher tumor or plasma expression levels than nontumor controls. Negative; lower tumor or plasma expression levels than nontumor controls. Thus, poorer prognosis correlates with increased expression levels of oncogenes and decreased expression of tumor suppressors, as compared to healthy controls. Table adapted and improved from [61].

**Table 3:** Summary of tumor suppressor lncRNAs and onco-lncRNAs involved in NPC.

lncRNAs (Biomarkers)	Main Biological Function(s)	Method of Detection	Potential Function of lncRNAs
**Tumor suppressor lncRNAs**			
LET	Inhibits the proliferation of NPC cells and induces cell apoptosis; transcriptional repressed by EZH2-mediated H3K27 histone methylation on the LET promoter	qRT-PCR [125]	-
LINC00312-NAG7	Inhibits proliferation, induces apoptosis, and cell invasion	In situ hybridization	Diagnosis, Prognosis [100]
MEG3	Repression of cell proliferation, colony formation, induction of cell cycle arrest and tumorigenicity in vitro and in vivo	Bisulfite sequencing, MS-PCR [126]	-
LINC0086	Inhibits cell proliferation and promotes apoptosis. Upregulation of LINC0086 decreased miR-214 expression	In situ hybridization, qRT-PCR [127]	-
LINC01133	Inhibits cell proliferation, invasion, and migration both in vitro and in vivo	qRT-PCR	Prognosis [101]
ENSG00000230489 – VAV3-AS1	Downregulation of VAV3-AS1 leads to interference with natural killer cell-mediated cytotoxicity	qRT-PCR	Diagnosis [128]
**Onco-lncRNAs**			
LINC01385	Promotes cell proliferation via miR-140-3p/Twist1 signaling pathway	qRT-PCR	Prognosis [120]
UCA1	Promotes cell progression by modulating miR-124-3p/ITGβ1 axis	qRT-PCR	Diagnosis [119]
LINC00460	Aggravates invasion and metastasis by targeting miR-30a-3p/Rap1A axis	qRT-PCR	Prognosis [118]
LINC01503	Facilitates proliferation and metastasis via SFPQ-FOSL1 axis	qRT-PCR	Prognosis [115]
MACC1-AS1	Promotes cell stemness via suppressing miR-145-mediated inhibition on SMAD2/MACC1-AS1 axis	In situ hybridization, qRT-PCR	Diagnosis [129]
LINC00346	Contributes to cisplastin resistance by repressing miR-342-5p	qRT-PCR	Prognosis [122]
HOTTIP	Promotes cell proliferation, migration and invasion by inhibiting miR-4301	qRT-PCR	Prognosis [106]
DRAIC	Acts as a miR-122 sponge to facilitate cell proliferation, migration, and invasion via regulating SATB1	qRT-PCR	Prognosis [111]
ENSG00000227084	Interferes with Rap1 signaling pathway	qRT-PCR	Diagnosis [128]
PXN-AS1-L	Promotes cell proliferation, migration, and invasion in vitro and in vivo. It promotes NPC malignancy by upregulating SAPCD2 via direct RNA–RNA interaction	qRT-PCR	Prognosis [117]
CASC15	Promotes cell proliferation and metastasis by downregulating miR-101-3p	qRT-PCR	Prognosis [116]
NPCCAT1	Promotes cell growth, migration in vitro, and in vivo. Upregulates YY1 protein to promote NPC progression	qRT-PCR [112]	-
PVT1	Inhibits cell proliferation, induces apoptosis	In situ hybridization, qRT-PCR	Prognosis [105]
ANRIL	Inhibits cell proliferation in vitro and in vivo. SOX2-ANRIL-β-catenin plays a role in NPC proliferation	qRT-PCR	Prognosis [113]
XIST	XIST upregulates E2F3 in part through “sponging” miR-34a-5p	qRT-PCR [109]	-
ROR	Suppress p53 signal pathway, promotes proliferation, migration, and chemoresistance	qRT-PCR	Diagnosis, Prognosis [108]
AFAP1-AS1	Inhibits AFAP1 protein expression and affects the expression of several small GTPase family members and molecules in the actin cytokeratin signaling pathway	qRT-PCR	Diagnosis, Prognosis [130]
HOTAIR	Promoted angiogenesis through directly activating the transcription of angiogenic factor VEGFA as well as through GRP78-mediated upregulation of VEGFA and Ang2 expression	In situ hybridization, qRT-PCR	Diagnosis [131], Prognosis [107]
HNF1A-AS	Promotes proliferation, migration, and EMT	qRT-PCR [110]	Diagnosis
H19	Inhibits E-cadherin expression and promotes invasion via the miR-630/EZH2 pathway	qRT-PCR [114]	-
NEAT1	Regulates EMT phenotype and radioresistance by modulating the miR-204/ZEB1 axis	In situ hybridization, qRT-PCR	Diagnosis, Prognosis [124]
MALAT1	Regulates CSC activity and radioresistance by modulating miR-1/slug axis	In situ hybridization, qRT-PCR [123]	Diagnosis, prognosis [130]
LINC00319	Promotes cell growth in vitro. LINC00319 contributed to NPC progression by regulating miR-1207-5p/KLF12 signal pathway	qRT-PCR	Prognosis [121]

MS-PCR; methylation-specific polymerase chain reaction, qRT-PCR; reverse transcriptase real-time polymerase chain reaction.

**Table 4:** Major samples used in detecting promising NPC biomarkers.

Type of Samples	Frequency of Oncogenes Involved (%)	Frequency of Tumor Suppressors Involved (%)
NPC primary biopsy	EBV-LMP1 (62.5%) [24] EBV-BARF1 (13.3%) [24] miR-141 [136] lncRNA PVT1 (64%) [105] lncRNA NPCCAT1 [112] lncRNA CASC15 [116] lncRNA PXN-AS1-L [117] lncRNA HOTTIP [106] lncRNA LINC00346 [122] lncRNA LINC01503 [115] lncRNA LINC00460 [118] lncRNA UCA1 [119] lncRNA ANRIL [113] lncRNA H19 [114] lncRNA MALAT1 [123] lncRNA NEAT1 [124] lncRNA LINC01385 [120] lncRNA HNF1A-AS [110]	p16 (23–66%) [32,33,34] p15 (50–80%) [38,39] RASSF1A (46–67%) [38,39] DAP-kinase (75–77%) [38,43] RIZ1 (60%) [137] CDH1 (50%) [39,46] E-cadherin (52–65%) [38,46] HIN-1 (77%) [48] MGMT (28%) [39] MLH1 (40%) [39] DLC1 (79%) [138] SOX11 (67.4%) [50] 14-3-3 sigma (84%) [51] PTEN (82.2%) [139] miR-9 [140] lncRNA LINC00312-NAG7 (51.4%) [100]
Formalin-fixed paraffin-embedded sample	ABL1 (1.6%) * AKT1 (0%) * AKT2 (0%) * BRAF (0.8%) * CDK (0%) * EGFR (0.8%) * ERBB2 (0%) * FGFR1 (0%) * FGFR3 (0%) * FLT3 (0%) * HRAS (0.8%) * JAK2 (0%) * KIT (3.3–33%) [18,141] KRAS (0%) * MET (0%) * NRAS (4.1%) * PDGFRA (1.6%) * PIKC3A (4.9–62.96%) [18,142] RET (0%) * ROCK1 (28.4%) [142] EBV-miR-BART8-3p (52%) [143] miR-3182 (51%) [143] miR-18a (71.1%) [144] miR-149 (82.4%) ** miR-141 (52.9%) ** miR-205 (94.1%) ** miR-196a (88.2%) ** miR-149 (82.4%) ** miR-183 (64.7%) ** miR-224 (58.8%) ** miR-210 (58.8%) ** miR-136 (47.1%) ** miR-200c (64.7%) ** lncRNA MACC1-AS1 [129] lncRNA HOTAIR [107]	p16 (5%) [145] miR-150 (82.4%) **
NP brushing	BARF1 [146] EBV-miR-BART1–5p [147] EBV-miR-BART5 [147] EBV-miR-BART6-5p [147] EBV-miR-BART17-5p [147]	WIF1 (61.2%) [49] p16 (46.4 –66%) [35,49] RASSF1A (39.3–75.5%) [35,49] DAP-kinase (79.2%) [49] RIZ1 (56.6%) [49] DLC1 (76.9%) [49]
NP swabs	LMP1 [148]	p16 (17%) [38] RIZ1 (37%) [137] E-cadherin (27%) [38]
M&T rinsing fluid	-	p16 (17%) [38] RIZ1 (30%) [137] E-cadherin (43%) [38]
Plasma	EBV-miR-BART7 [98] EBV-miR-BART13 [98] miR-21 [60]	p16 (42%) [31] RIZ1 (23%) [137] miR-9 [87]
PBMCs	PIKC3A [149] TP53 [149]	RIZ1 (10%) [137]
Exosomes	HIF1α [150] LMP1 [151]	DLEC1 (25%) UCHL1 (64.9%) miR-9 [152]

* Data from reference [18] ** Data from reference [153].
